# Retraction Note: Impact of weight loss diet associated with flaxseed on inflammatory markers in men with cardiovascular risk factors: a clinical study

**DOI:** 10.1186/s12937-016-0165-x

**Published:** 2016-06-07

**Authors:** Roberta Soares Lara Cassani, Priscila Giacomo Fassini, Jose Henrique Silvah, Cristiane Maria Mártires Lima, Júlio Sérgio Marchini

**Affiliations:** 1 Institute of Nutrition, Itu, São Paulo Brazil; 2 Department of Medicine, Division of Medical Nutrition, Ribeirão Preto Medical School, University of São Paulo, Avenida Bandeirantes, 3900 Bairro Monte Alegre, Ribeirão Preto, São Paulo CEP: 14049-900 Brazil

The Editor is retracting this article [1] because of concerns raised after publication with respect to the methods and the statistical analysis [2] which the authors have not been able to adequately address [2]. We apologise to all affected parties for the inconvenience caused. All authors support this retraction.
